# Oral Acantholytic Squamous Cell Carcinoma: A Rare Case of Mandibular Alveolar Ulceration

**DOI:** 10.7759/cureus.62340

**Published:** 2024-06-13

**Authors:** Rajoshee R Dutta, Tanishq Kumar, Kaushal Charan Pahari, Arihant Singh, Bhushan Madke

**Affiliations:** 1 Medicine, Jawaharlal Nehru Medical College, Datta Meghe Institute of Higher Education and Research, Wardha, IND; 2 Maxillofacial Surgery, SB Aesthetics, Gurugram, IND; 3 Dermatology, Venereology, and Leprosy, Jawaharlal Nehru Medical College, Datta Meghe Institute of Higher Education and Research, Wardha, IND

**Keywords:** modified radical neck dissection, mandibular alveolar ulcer, oral cancer, angiosarcoma, squamous cell carcinoma, acantholytic squamous cell carcinoma

## Abstract

Acantholytic squamous cell carcinoma (ASCC) is an atypical form of squamous cell carcinoma (SCC). Although it is well known that ASCC typically appears in sun-exposed regions of the face and neck, oral cavity cases are incredibly rare. In this case report, we present a rare case of a 50-year-old male who developed an ulcer on his right mandibular alveolus, diagnosed with ASCC post-biopsy. On histopathological analysis, acantholytic cells with a pseudo-glandular appearance were observed. Subsequently, the tumor was resected by modified radical neck dissection with a split-thickness graft. The patient responded well to surgery and had no complications post-surgery.

## Introduction

Acantholytic SCC (ASCC) is an extremely unusual form of SCC that presents as a skin-colored nodular structure. It is also known as adenomatoid squamous cell carcinoma (SCC) or adenoacanthoma. It is distinguished by a characteristic SCC pattern, as well as the presence of dyskeratotic cells, pseudo-glandular structures, and significant acantholysis surrounding islands of parts of the tumors [1]. Such a breakdown of cell adherence, known as acantholysis, occurs as a result of desmosomal proteins being absent. This results in cells that may look highly irregular, enlarged, or have multiple nuclei. ASCC occurring in sun-exposed skin areas appears to exhibit a higher incidence of relapse or potential metastatic invasion compared to typical SCC [2]. Intraoral ASCCs are believed to be more aggressive, potentially leading to a poor prognosis, necessitating a multidisciplinary treatment approach [3]. Less than 60 instances of ASCC have been documented in global literature so far [1,4]. This case report presents a rare case of ASCC in the oral mucosa without skin adnexal structures. The manifestation of ASCC in this case is unique due to minimal sunlight exposure. Therefore, there is no apparent triggering factor for its precipitation. 

## Case presentation

**Figure 1:**
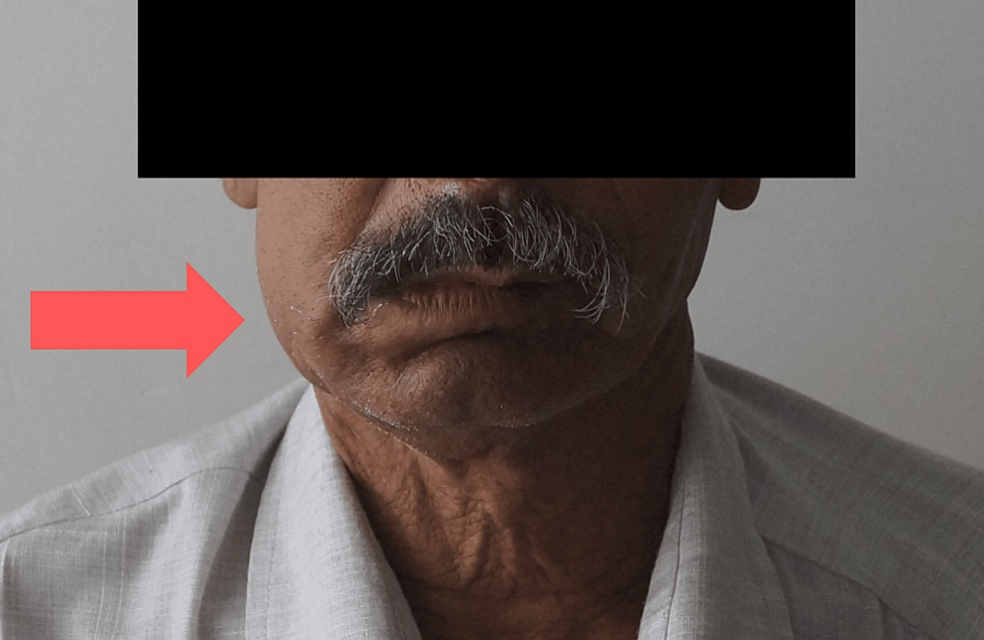
Pre-operative image of the patient where swelling is apparent on the right mandibular side

A 50-year-old man came to the Maxillofacial and Oral Surgery Department of a Tertiary Care Facility with a chief complaint of swelling in the lower right back teeth area for the previous six months. Figure 1 shows a pre-operative image of the patient with a right-sided swelling.

The personal history of the patient showed that he had been a tobacco chewer for 10 years. He has also taken one tablet of buprenorphine daily for one year to quit his habit of doda post. Clinical examination findings were not noteworthy except for the lesion. The overall health of the patient was normal, and there were no relevant issues found in his medical history.

**Figure 2:**
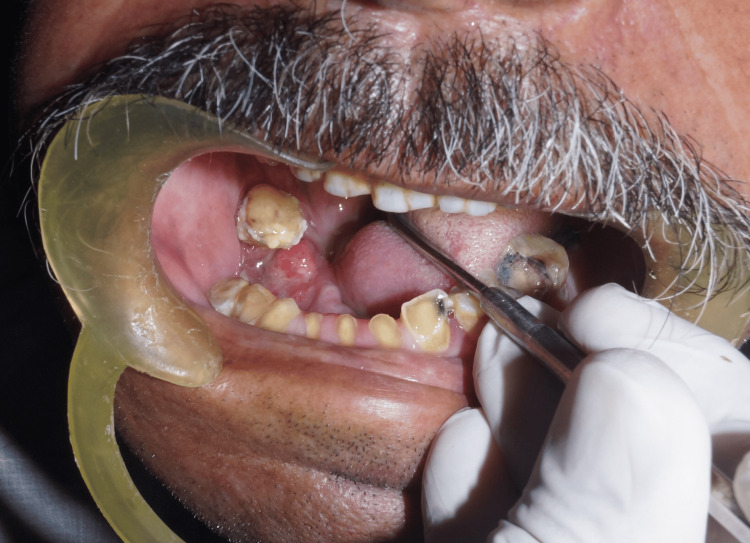
Pre-operative image of the patient in which ulcerated growth extends from the alveolar crest to vestibule of the right side

An extraoral examination revealed gross facial asymmetry with swelling on the right mandibular side. On palpation, there was tenderness present on the right side of the mandible. The submandibular lymph nodes were tender, enlarged, firm in consistency, mobile, and approximately 1 cm in size. Intra-oral examination revealed an ulcer of approximately 3 x 1.5 cm in size seen on quadrant 4, spanning from #42 to #47. The ulcerated growth extended from the alveolar crest to the vestibule of the right side shown in Figure 2.

The base of the ulcer was yellow, and the margins were rolled. On palpation, the lesion was tender, and fibrous bands were felt on the right side. A provisional diagnosis of “SCC of the right lower alveolus” was established based on the clinical findings and the patient’s medical history. 

Investigations

An incisional biopsy was performed for histopathological analysis. The tissue was processed routinely, and hematoxylin and eosin (H&E) stain was used for staining following fixation in a 10% formalin solution. 

**Figure 3:**
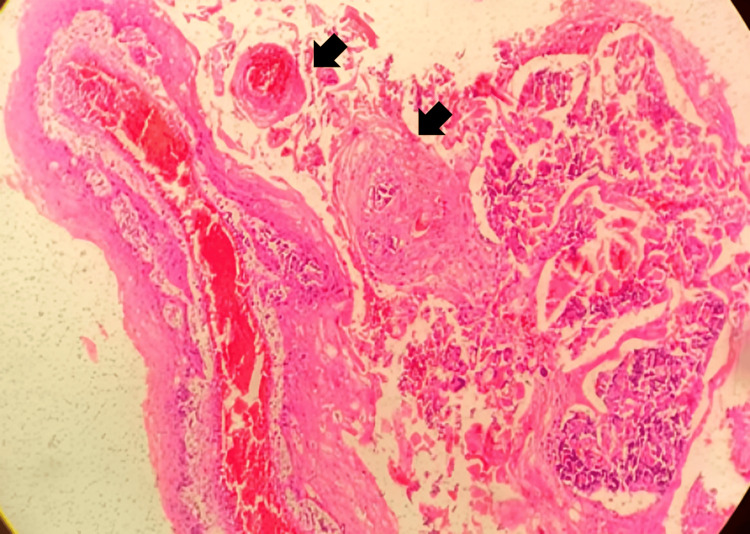
H&E section showing acantholytic cells with atypical dyskeratosis and bizarre morphology with multinucleation

On microscopic examination, the H&E section shows the presence of tubular structures along with lobules and nests of malignant epithelial cells with loss of intracellular adhesion in the center revealing an appearance similar to a pseudogland. There were floating acantholytic cells with atypical dyskeratosis in the central areas and bizarre morphology with multinucleation shown in Figure 3.

A few acantholytic tumor cells also showed dense eosinophilic cytoplasm and vesicular nuclei. Some discohesive cells showed scanty cytoplasm. The tumor islands and cells stained negative on periodic acid Schiff (PAS) and Mucicarmine. The underlying dense fibrous connective tissue shows chronic inflammation and abundant muscle, tissue, and bone. There is the presence of numerous vessels and anastomosing channels with dilated and congested vascular spaces. Based on clinical and histopathological examination, findings were suggestive of “Pseudovascular ASCC.” 

Therapeutic interventions

Surgical intervention by wide local excision and a modified radical neck dissection (MRND) Type 2 with reconstruction of the pectoralis major muscle flap with a split-thickness skin graft from the thigh was done. As it is difficult to rule out lymphatic metastasis based on histopathology, neck dissection is performed.

**Figure 4:**
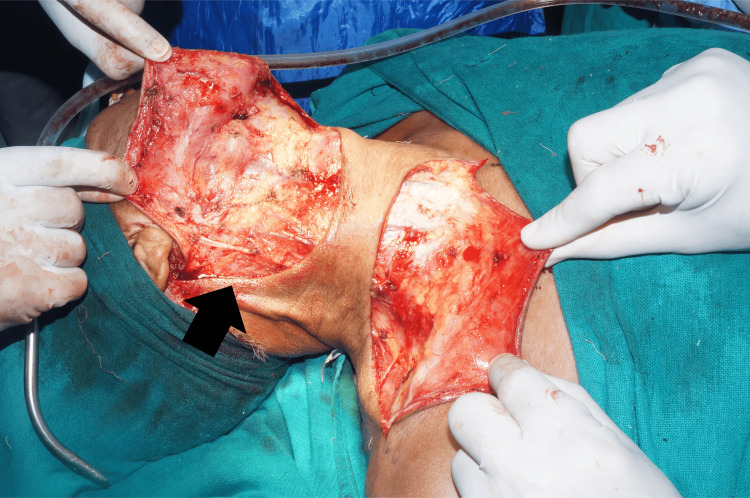
Incision followed by flap elevation

**Figure 5:**
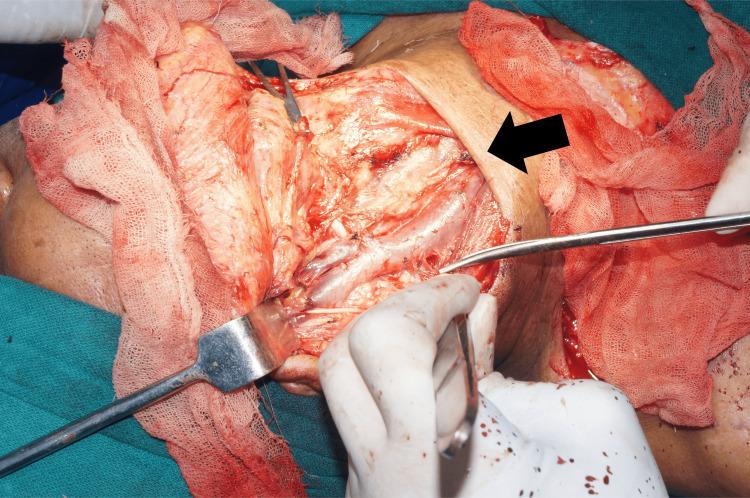
Intraoperative image of modified radical neck dissection type 2

The excisional biopsy confirmed the tumor through histopathological examination, revealing clear margins and no involvement of neck nodes or signs of lymphatic metastasis. There was no sign of recurrence on follow-up visits, and the operated site healing was satisfactory. Figures 4, 5 show intraoperative images of the surgery.

**Figure 6:**
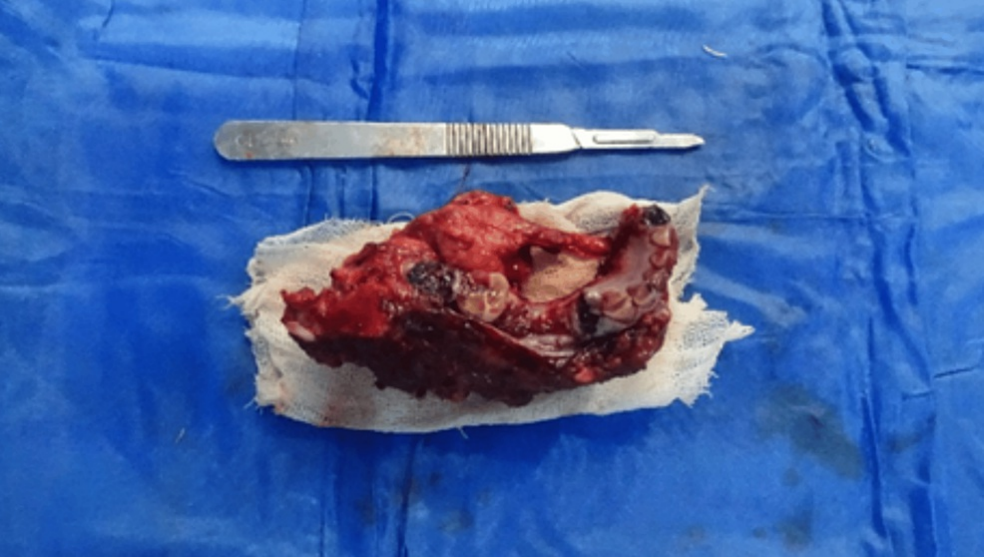
Excised specimen

Figure 6 shows an image of the excised lesion post-surgery.

## Discussion

Sun-exposed parts are the most prevalent sites for ASCC, a histological subtype of SCC. It is rarely observed on the upper aerodigestive tract’s mucosal surfaces. This SCC variant was initially reported in 1947 [5].

Alternative names for ASCC include adenoacanthoma, pseudoangiosarcomatous carcinoma, angiosarcoma-like SCC, and pseudo-glandular SCC. It is distinct from typical SCC in terms of its aggressiveness and histologic characteristics. On microscopy, the neoplastic epithelium of the tumor exhibits cystic degeneration, resulting in the development of pseudo-glandular structures containing acantholytic cells and a distinct alveolar pattern. This finding is linked to a decrease in cell adhesion in the tumor nest centers due to immunohistochemistry expression of E-cadherin being lost [6]. The sixth decade has been estimated to have the highest incidence of ASCC. There may be a relationship between this variant of SCC and prior ionizing radiation exposure [7]. It rarely develops intraorally and mostly affects the lips [8].

Despite being entirely tumor types, angiosarcoma and ASCC share intra-tumoral gaps as common histological characteristics. Anastomosing gaps and channels form in ASCC in a manner that is similar to angiosarcoma. It is interesting to note that both tumor entities have similar clinical characteristics. Both angiosarcoma and ASCC are rapidly expanding, eruptive lesions associated with a bad prognosis from a macroscopic perspective [9]. ASCC exhibits a male predisposition, similar to other SCCs, although oral angiosarcoma has no documented sex preference. Angiosarcoma and ASCC exhibit overlaps in vascular differential markers and cytokeratin expression in addition to having the same clinical characteristics and a comparable histological pattern when stained similarly. Angiosarcoma’s synthesis of Fli-1 and ASCCs cytoplasmic immunoreaction for the γ2-chain of ln-5 are their respective distinctive characteristics [10].

ASCC is identified by negative staining for mucin and the lack of glandular structures, which are characteristics of adenocarcinomas, especially adenosquamous carcinomas. As ASCC has glandular spaces and fibrin which are often mistaken for mucin, it can resemble adenoid cystic carcinomas. On the other hand, in ASCC, glandular spaces frequently seem angular, and epithelial mucin is not visible in mucin stains. Typically, foci associated with conventional SCC accompany ASCC which helps in making an appropriate diagnosis. Additionally, only focal glandular forms are present in adenosquamous carcinomas throughout the entirety of the lesion. Mucoepidermoid carcinoma (MEC) may be considered if a squamous element is identifiable, and the lesions are misinterpreted as glandular lesions. Mucin and rounded glandular areas are readily identifiable in the low and intermediate stages of MEC. Additionally, there rarely exists an excess of glandular forms in high-grade MEC [11].

This case of ASCC is particularly rare, as it manifests in the oral mucosa, as opposed to the more prevalent occurrence in sun-exposed parts of the skin. The patient has no history of ionizing radiation, which is frequently linked to the development of such carcinomas. Histopathologically, the tumor had the typical pseudo-glandular appearance of acantholytic cells, a hallmark of ASCC, and can complicate differentiation. This case report stresses the significance of a thorough histological examination and the relevance of immunohistochemical staining in identifying ASCC. Furthermore, it investigates the therapeutic consequences, noting that, while ASCC can be aggressive, early identification and appropriate surgical interventions can considerably improve patient outcomes.

## Conclusions

In conclusion, oral cavity-specific ASCC is a unique histological variant of SCC. The global epidemiology of ASCC remains poorly documented with less than 60 case reports so far, highlighting the necessity for comprehensive research in this area. Our case report illustrates the rarity and incidence of ASCC, emphasizing the importance of histopathological investigations for its diagnosis. Oral ASCC may exhibit pseudo-glandular and pseudo-vascular morphology. It is crucial to differentiate ASCC from adenosquamous carcinoma, where the adenocarcinoma element tests positive for mucin. The prognosis of mucosal lesions remains controversial due to the limited number of reported cases of intraoral ASCC, which hinders a clear understanding of its biological behavior and prognosis. 
